# Determining Antigen Specificity of Human Islet Infiltrating T Cells in Type 1 Diabetes

**DOI:** 10.3389/fimmu.2019.00365

**Published:** 2019-03-08

**Authors:** Maki Nakayama, Aaron W. Michels

**Affiliations:** Barbara Davis Center for Childhood Diabetes, University of Colorado School of Medicine, Aurora, CO, United States

**Keywords:** diabetes, autoimmunity, HLA, insulin, T cells

## Abstract

Type 1 diabetes, the immune mediated form of diabetes, represents a prototypical organ specific autoimmune disease in that insulin producing pancreatic islets are specifically targeted by T cells. The disease is now predictable in humans with the measurement of type 1 diabetes associated autoantibodies (islet autoantibodies) in the peripheral blood which are directed against insulin and beta cell proteins. With an increasing incidence of disease, especially in young children, large well-controlled clinical prevention trials using antigen specific immunotherapy have been completed but with limited clinical benefit. To improve outcomes, it is critical to understand the antigen and T cell receptor repertoires of those cells that infiltrate the target organ, pancreatic islets, in human type 1 diabetes. With international networks to identify organ donors with type 1 diabetes, improved immunosequencing platforms, and the ability to reconstitute T cell receptors of interest into immortalized cell lines allows antigen discovery efforts for rare tissue specific T cells. Here we review the disease pathogenesis of type 1 diabetes with a focus on human islet infiltrating T cell antigen discovery efforts, which provides necessary knowledge to define biomarkers of disease activity and improve antigen specific immunotherapy approaches for disease prevention.

## Introduction

**Figure 1:**
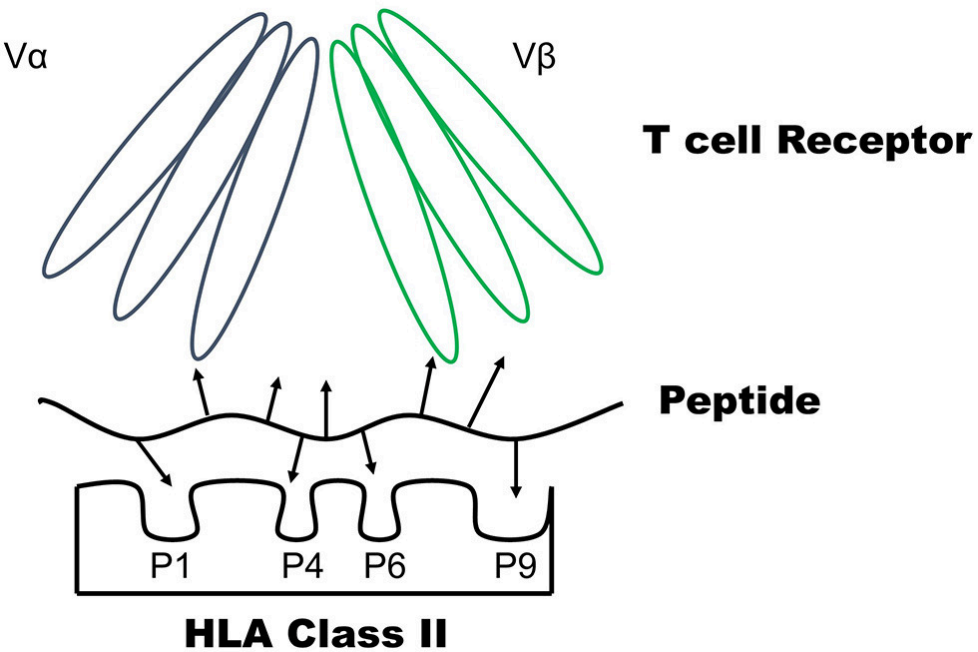
The trimolecular complex consists of a CD4 T cell receptor–Peptide–HLA class II molecule. The peptide binding groove of the HLA class II molecule consists of pockets (P1, P4, P6, and P9) which bind amino acid side chains to anchor a peptide. The arrows of the peptide represent amino acid side chains. The T cell receptor is comprised of alpha and beta chains with three complementary determining regions (CDR) each. The T cell receptor interacts with both peptide amino acid side chains and those located on the HLA molecule to become activated.

Type 1 diabetes is a chronic autoimmune disorder that results from the tissue specific destruction of insulin producing beta-cells within pancreatic islets (1, 2). Human leukocyte antigen (HLA) genes, especially the class II DQ and DR alleles, confer significant disease risk (3–5). In addition to T1D risk genes, there are yet to be identified environment factors that lead to a loss of tolerance to insulin and other beta cell proteins. It is now appreciated that T1D develops in stages prior to the clinical onset of symptoms and these stages are defined with the presence of islet autoantibodies (6), those directed against insulin, glutamic acid decarboxylase (GAD), islet antigen 2 (IA-2), and zinc transporter 8 (ZnT8). From prospective birth cohort studies, if an at-risk child has two or more islet autoantibodies, there is ~85% chance of developing T1D within 15 years and a nearly 100% lifetime risk for disease development (7). With the ability to stage the disease process and an increasing incidence over the last two decades (8), a number of largescale immune intervention trials have been completed trying to delay or prevent the onset of clinical disease. Many of these trials have used formulations of insulin (subcutaneous, intranasal, oral, and intradermal) as an antigen specific therapy (9–13). Unfortunately, the trials have been of limited clinical benefit and there is a need for safe and specific therapies to prevent the onset of T1D (14). We believe understanding the immunology within the target tissue, pancreatic islets, will lead to improved markers of disease activity and therapies to delay T1D onset (15). This has been challenging due to the anatomic location of the pancreas and dual function as both an exocrine and endocrine organ. However, recent efforts and advances in technology have made the study of pancreatic islets from recent onset T1D organ donors possible. Here we review the progress made in understanding the reactivity of islet infiltrating CD4 T cells with a focus on the trimolecular complex of T cell receptor–peptide–HLA molecule (Figure 1).

## Stages of T1D Development

At the current time, a significant amount of understanding surrounds the natural history of T1D development. Much of our understanding comes from both animal models of disease development and birth cohort studies that have followed genetically at-risk children (i.e., those with high risk HLA class II DQ and DR alleles) prospectively from birth to disease onset. These large epidemiology studies have taken place in the United States, Diabetes AutoImmunity Study in the Young (DAISY) (16), and Europe, including the Type 1 Diabetes Prediction and Prevention Study (DIPP) in Finland (17), and BABYDIAB studies in Germany (18). All three studies measured islet autoantibodies from the peripheral blood at regular time intervals (every 3–6 months) and showed that the presence of two or more islet autoantibodies marks the start of T1D, as nearly all of these genetically at risk children and adolescents develop clinical T1D, indicated by elevated blood glucose, insulin deficiency, and the need for exogenous insulin treatment. Notably, islet autoantibodies are markers of a disease state (islet autoimmunity) but not disease activity as antibodies can be present for years prior to clinical T1D presentation (19). The preclinical phase of T1D is now staged by the presence of islet autoantibodies and beta cell function, as measured by an oral glucose tolerance test (6). Stage 1 is the presence of two or more islet autoantibodies with no dysglycemia, Stage 2 encompasses those with autoantibodies and metabolic abnormalities, and Stage 3 is prototypical new-onset T1D in which an individual meets diagnostic blood glucose criteria for diabetes and the presence of islet autoantibodies (20). This staging paradigm leads to T1D being predictable and the ability to perform clinical intervention trials prior to a significant loss of beta cell function, with these trials previously reviewed (21).

Significant insights into disease pathogenesis come from the studying of animal models of autoimmune diabetes (22). The non-obese diabetic (NOD) mouse is a spontaneous model of autoimmune diabetes and shares many similarities with human disease (23). Both have major histocompatibility (MHC) class II genes that confer disease risk, NOD mice develop autoantibodies to insulin prior to hyperglycemia, and immune cells including T cells infiltrate the pancreatic islets (termed insulitis) in these mice. Disease specific T cells from the islets and pancreatic lymph nodes reveal that insulin is a critical self-antigen in the disease process (24–29). A fragment of the insulin B chain, amino acids 9-23 (B:9-23), is a CD4 T cell epitope and mutation of a single amino acid within the B chain (B16 tyrosine to alanine) results in mice maintaining normoglycemia without insulitis (27). This is not the case for other beta cell antigens including GAD, islet antigen 2 and glucose-6-phosphatase catalytic subunit-related protein (30–32) with the exception of chromogranin A, elimination of which results in significant protection from islet autoimmunity in NOD mice (33).

These insights from the NOD have translated to human disease in that insulin B:9-23 reactive T cells have been identified within the islets of recent onset organ donors and in the peripheral blood of T1D patients (34–38). Notably insulin B:9-23 has an identical amino acid sequence in mouse and humans. Further support for insulin as a key self-antigen in a subset of T1D patients comes from the TEDDY study, The Environmental Determinants of Diabetes in the Young, which is an international multicenter prospective study evaluating environmental factors that may lead to the development of autoantibodies (islet autoimmunity) and eventual T1D (39). It is becoming appreciated that genetically at-risk children present with a different initial islet autoantibody that are correlated to HLA-DQ-DR genotypes. The presence of HLA-DR4-DQ8 haplotype (DR and DQ are in linkage disequilibrium on chromosome 6) is associated with insulin autoimmunity, while the DR3-DQ2 haplotype may reflect initial autoimmunity to GAD (40). These important findings indicate: (1) T1D risk genes are strongly associated with markers of an adaptive immune response to self-antigen and (2) there is disease heterogeneity such that subsets of patients may be defined by the presence of given HLA genes.

Another animal model of autoimmune diabetes that has been instrumental in understanding mechanisms of disease development is the BioBreeding rat. In these models there are diabetes prone animals that develop spontaneous islet infiltration, and separately diabetes resistant rats in which diabetes is induced with a viral infection or other stimulus of innate immune system activation (41). The use of an environmental trigger, such as a viral infection, may mimic an environmental exposure needed to induce human islet autoimmunity, and eventual T1D in a genetically susceptible individual.

Animal models unfortunately have not been as robust of a model for clinical therapeutics (42). The potential reasons for this are many, including dose, route, and timing of an intervention in the disease process. For example, antigen specific therapies tend to work well to prevent NOD diabetes onset when administered early in the disease process (e.g., before the development of insulin autoantibodies) but become less effective as the disease progresses (43). In contrast, anti-CD3 monoclonal antibodies are able to delay disease onset when administered later in the NOD disease course (42). Just as there are different animal models of T1D with spontaneous and inducible disease processes, human T1D is likely not a single disease. There is disease heterogeneity and the need exists to understand different subtypes of the condition, i.e., those that have a distinct pathophysiologic mechanism leading to T1D development (44), to improve the development of disease modifying therapies for T1D. As HLA class II genes confer genetic risk and function to present processed antigens to CD4 T cells, understanding this trimolecular complex (Figure 1) within the target tissue (pancreatic islets) in human disease is critical for disease prevention and reversal (45, 46).

## Network for Pancreatic Organ Donors (nPOD)

The Network for Pancreatic Organ Donors (nPOD, http://www.jdrfnpod.org/) was established in 2007 to address the gap in the field to study the target organ in autoimmune T1D (47). Tissues from organ donors are collected and distributed to investigators for the study into the pathogenesis of human T1D. To date over 150 cases have been collected from T1D patients, over 150 non-diabetic donors and several dozens with autoantibodies but no clinical diabetes. This large and collaborative consortium has provided the frame work to study the human pancreas, islets embedded within the pancreas, pancreatic lymph nodes, and spleen in those with and without disease. Many insights have been gleaned from a decade of studies and recently reviewed (48, 49).

Prior to the efforts of nPOD, most of the knowledge regarding T cell reactivity came from the study of rare antigen specific cells in the peripheral blood with multiple groups cloning islet reactive T cells (35, 36, 50–53). Outside of the peripheral blood, Kent and colleagues cloned insulin A chain reactive CD4 T cells from the pancreatic lymph nodes of an individual with established disease (54). However, in the last 3 years, three independent laboratories have reported on the antigen specificity of human islet derived CD4 T cells from recent onset T1D organ donors.

## Antigen Repertoire of Human Islet Infiltrating T Cells

**Figure 2:**
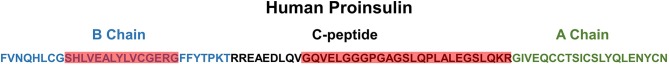
Amino acid sequence of human proinsulin with shaded areas representing CD4 T cell epitopes recognized by islet derived T cells. T cells obtained from the insulitis lesions of recent onset T1D organ donors respond to epitopes within the shaded area of the insulin B chain, the B:9-23 region, and C-peptide including the amino acid sequence GQVELGGG which forms a portion of hybrid insulin peptides. These islet derived T cells are predominantly activated by peptides presented by HLA-DQ8.

In 2015, Mannering and colleagues reported the first results of cloning islet resident CD4 T cells from a deceased T1D organ donor (19 year-old with 3 years of T1D) (55). The donor had residual insulin staining within islets and hand-picked islets were cultured under conditions to promote T cell growth. Outgrowths of T cells were apparent and these cells were then single cell sorted using flow cytometry. Clones were established and then tested for reactivity to overlapping peptides of proinsulin and 26 peptides derived from other islet autoantigens–GAD65, IA-2, IGRP, and ZnT8. Epstein Barr Virus (EBV) transformed B cells from the donor were used as antigen presenting cells (APCs) in these T cell stimulation assays. Remarkably, 14 out of 53 tested CD4 T cell clones (26%) responded to six overlapping peptides within the C-peptide region of proinsulin (55) (Figure 2). The HLA restriction element was predominantly DQ8 and one clone responded to a C-peptide fragment presented by DQ8 trans. HLA-DQ8 (DQA^*^03:01, DQB^*^03:02) is present in ~60% of all T1D patients and those at risk and confers an odds ratio for disease development of 6.5–11 (3). HLA-DQ2 (DQA^*^05:01, DQB^*^02:01) is also present in about 1/3 T1D patients, and trans dimers can be formed where the beta alpha chain of DQ2 pairs with the beta chain of DQ8 (DQ8 trans: DQA^*^05:01, DQB^*^03:02), which may present unique epitopes to autoreactive CD4 T cells in T1D (53, 56, 57).

Using a similar approach, Kent and colleagues cloned CD4 T cells from hand-picked islets of nine recent onset organ donors with T1D (58). In addition to growing T cell lines directly from islets, dispersed islets were stained, flow sorted for CD4 and CD8 cells and then expanded to establish *ex vivo* T cell lines. Fifty of the CD4 T cell lines were tested for antigen specificity using autologous EBV transformed B cells as APCs and a large panel of known or putative islet antigens including some which were post-translationally modified. Antigen specificity was identified in 17/50 (34%) of the tested T cell lines with a wide array of antigens represented including some which may represent post-translationally modified peptides (58, 59). These T cell lines predominantly secreted inflammatory cytokines such as IFN-γ, TNF-α, and IL-2 in response to their cognate peptide/HLA.

We used an alternate but complementary approach to study islet infiltrating T cells from three recent onset T1D organ donors, all of whom had insulitis and the T1D risk HLA-DQ8 allele (37). Hand-picked islets underwent short-term culture (~3–4 days) followed by single cell flow sorting for CD4 and CD8 cells. Instead of attempting to expand the single T cells, we sequenced the alpha and beta chains of the TCR of each individual cell. This provided insights into the diversity of the TCR repertoire of islet infiltrating T cells. We were able to isolate hundreds to thousands of T cells from 500 to 1,500 islet equivalents. CD8 T cells were more clonally expanded in these donors as 1/3 to 1/2 of all full length receptors were detected more than twice in the same donor (37). For CD4 T cells, only 15–20% of the sequences were detected more than twice from two of the donors (37). While none of the identical sequences were shared between the three patients, it could due to the limited number of cases studied and further efforts may reveal public TCRs shared across patients.

To test antigen specificity, the TCR sequences from CD4 T cells were transduced into an immortalized TCR null cell line, thus making a single TCR transductant, and screened against overlapping preproinsulin peptides and other characterized islet antigens derived from peripheral blood T cell reactivity of patients. These TCR transductants are readily expanded in culture and provide a robust reagent to determine antigen specificity as these cells secrete IL-2 when the TCR engages cognate peptide/HLA (60). From 85 selected CD4 TCR transductants, 3 responded to peptides within proinsulin (37). Two TCRs from two separate donors responded to insulin B:9-23 presented by DQ8 and one TCR responded to C-peptide 19-35 presented by DQ8 trans (Figure 2). Importantly, the insulin B:9-23 responding T cells also responded to whole islets as antigen when presented by APCs bearing DQ8. Notably, reactivity to the DQ8 trans epitope within C-peptide is identical to that reported by Mannering and colleagues in a separate patient (55). This raises the distinct possibility that there are common epitopes within proinsulin, insulin B:9-23 and C-peptide 19-35, that stimulate islet infiltrating CD4 T cells even after the clinical onset of T1D.

Further research is underway to characterize the antigen specificity of the remaining CD4 TCR transductants and in a similar manner the CD8 T cell specificities and their HLA restriction elements. It is notable that the majority of the T cell lines, clones, and transductants reported on to date have unknown antigen specificities. Developing high or moderate throughput screening systems will aid in this endeavor. As TCR transductants are engineered T cells, these cells are amenable to fluorescent reporter systems based upon TCR stimulation, thereby providing the ability to combine multiple T cells into a single well of a stimulation assay. The use of human islet extracts as a source of antigen, including those cultured with agents that induce beta cell “stress,” may provide tools to determine mechanisms by which autoantigens are formed in various disease conditions. Finally, combinatorial peptide libraries provide another avenue to define ligands for the islet derived T cell clones, lines, and transductants which have had success in other immune mediated diseases (61, 62).

## Implications for Studying Tissue Specific T Cells in T1D

For T1D, understanding the specificities and antigen receptor repertoire of those T cells within the target tissue will provide novel insights into disease pathogenesis and help to further dissect disease heterogeneity. Studying tissue specific T cells also provides an avenue to develop biomarkers to assess disease activity and monitoring immune responses to therapies. Islet autoantibodies mark a disease state in which the adaptive immune response has targeted pancreatic islets; however, these do not correlate to disease activity (i.e., insulitis and beta cell destruction). Conversely, T cells can cause tissue specific destruction and may provide markers of an active disease state, especially those cells that circulate in the peripheral blood. Of note, insulin B:9-23 reactivity has been detected in the islets of multiple T1D organ donors and peripheral blood of T1D patients (34–38). Further understanding the TCR repertoires across patients holds potential for developing a non-cell based biomarker assay as compared to traditional assays using fluorescent multimers (63, 64) and functional assays such as cytokine enzyme linked ImmunoSpot (ELISPOT) (65, 66). As immunosequencing technologies continue to advance, combined with machine learning algorithms, the potential exists to understand clonally expanded TCR repertoires and their ligands in disease states (67).

One of the major goals of studying human islet infiltrating T cells is to apply this knowledge to the development of therapies to prevent disease onset, induce tissue specific tolerance, and ultimately reverse the disease process. As mentioned previously, T1D does not represent a single disease pathogenesis and there are likely different mechanisms or pathways which lead to the immune system losing tolerance to insulin producing beta cells (44). It is important to understand these pathways and identify the different patient subsets or endotypes who share similar features to improve disease modifying therapies within T1D.

Antigen specific therapy has long held promise to both delete effector T cells and induce regulatory T cells to self-antigens (68). By understanding T cell reactivity within human T1D islets, it is our belief that antigen specific therapy can be better designed and subsets of patients identified with these T cell responses in their peripheral blood. It is conceivable that whole antigens (e.g., preproinsulin) or multiple relevant peptides need to be administered to patients with these reactivities. For example, a subset of patients may have dominant T cell responses directed toward insulin epitopes and benefit to proinsulin based antigen therapies, while other patients may have predominant GAD reactivity and would respond to GAD based antigen therapies. This rationale is in line with the findings from TEDDY indicating that a subset of children present with initial insulin autoimmunity or GAD autoimmunity based upon HLA genotype (40). An important caveat to mention is the fact that combination therapy will likely be needed based upon the stage of T1D treated (14). At new-onset T1D (Stage 3) induction therapy could be followed by antigen specific therapy in an attempt to induce antigen specific tolerance. Induction therapies could include a monoclonal antibody directed against CD2 or CD3 on T cells (69–72), CD20 on B cells (73), CD80/86 on APCs (74), or polyclonal anti-thymocyte globuliln (ATG) (75), which have all shown short-term ability to preserve residual beta cell function in clinical trials with new-onset T1D patients.

Another promising disease modifying therapy uses small “druglike” molecules to block self-antigens presented by HLA-DQ8 (76, 77). DQ8 is common in T1D, confers significant genetic risk and is actively involved in disease pathogenesis as the vast majority of islet derived CD4 T cells studied to date are activated by proinsulin peptides presented by DQ8 (58). Methyldopa (Aldomet), a clinically well-established oral medication used to treat hypertension in children and adults for >50 years (78), was discovered to bind the antigen-binding cleft of DQ8, and block peptide presentation and subsequent T cell activation to self-antigens (insulin and α-gliadin) (77). Remarkably, methyldopa did not alter a T cell response to an influenza epitope presented by DQ8. In a proof of concept clinical trial, recent onset T1D patients were genetically selected for DQ8 and administered methyldopa (www.clinicaltrials.gov NCT01883804). Methyldopa specifically blocked DQ8, not DR4 or DQ2, in these patients and lessened the inflammatory response of insulin specific T cells (77). As many autoimmune diseases are associated with specific HLA class II genes (79, 80), genetically selecting patients and treating with drugs to block self-antigen T cell activation by these HLA molecules has broad applicability to treating not only T1D but other autoimmune diseases.

## Conclusions

There is a need to study T cells within the target tissue, pancreatic islets, of T1D patients. This need is being met through collaborative research efforts such as the Network for Pancreatic Organ Donors (nPOD). Findings from independent laboratories are defining the components of the trimolecular complex, TCR-peptide-HLA, within human islet infiltrating CD4 T cells. This understanding provides a framework to understand disease heterogeneity and develop biomarkers of disease activity, which hold promise for disease monitoring and timing of therapeutic interventions. Ultimately, this understanding will aid in the design and development of improved therapies aimed at inducing tolerance to islet antigens and preventing T1D onset.

## Author Contributions

MN and AM both wrote and edited the manuscript.

### Conflict of Interest Statement

AM and MN are inventors on an issued patent, Compounds That Modulate Autoimmunity and Methods of Using the Same, licensed to ImmunoMolecular Therapeutics, LLC. AM is a scientific cofounder of ImmunoMolecular Therapeutics.
